# Nature of the Ligand-Centered Triplet State in Gd^3+^ β-Diketonate Complexes as Revealed by Time-Resolved EPR Spectroscopy and DFT Calculations

**DOI:** 10.1021/acs.inorgchem.1c01123

**Published:** 2021-10-06

**Authors:** Silvia Carlotto, Luca Babetto, Marco Bortolus, Alice Carlotto, Marzio Rancan, Gregorio Bottaro, Lidia Armelao, Donatella Carbonera, Maurizio Casarin

**Affiliations:** † Department of Chemistry, University of Padova, via F. Marzolo 1, 35131 Padova, Italy; ‡ Institute of Condensed Matter Chemistry and Technologies for Energy (ICMATE), National Research Council (CNR), c/o Department of Chemistry, University of Padova, via F. Marzolo 1, 35131 Padova, Italy; § Department of Chemical Sciences and Technology of Materials (DSCTM), National Research Council (CNR), Piazzale A. Moro 7, 00185 Roma, Italy

## Abstract

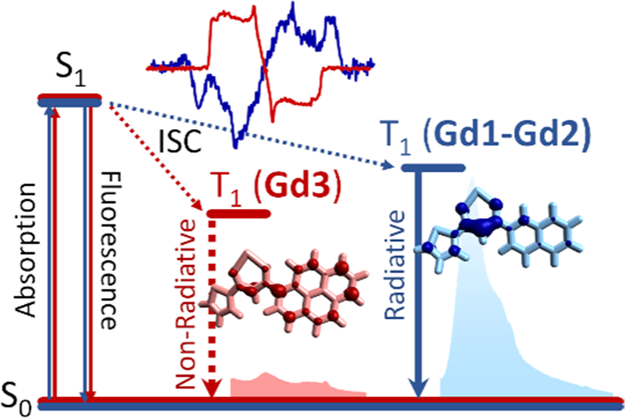

A series of Gd^3+^ complexes (**Gd1**–**Gd3**) with the general formula GdL_3_(EtOH)_2_, where L is a β-diketone ligand with polycyclic aromatic hydrocarbon substituents of increasing size (**1**–**3**), was studied by combining time-resolved electron paramagnetic resonance (TR-EPR) spectroscopy and DFT calculations to rationalize the anomalous spectroscopic behavior of the bulkiest complex (**Gd3**) through the series. Its faint phosphorescence band is observed only at 80 K and it is strongly red-shifted (∼200 nm) from the intense fluorescence band. Moreover, the TR-EPR spectral analysis found that triplet levels of **3**/**Gd3** are effectively populated and have smaller |*D*| values than those of the other compounds. The combined use of zero-field splitting and spin density delocalization calculations, together with spin population analysis, allows us to explain both the large red shift and the low intensity of the phosphorescence band observed for **Gd3**. The large red shift is determined by the higher delocalization degree of the wavefunction, which implies a larger energy gap between the excited S_1_ and T_1_ states. The low intensity of the phosphorescence is due to the presence of C–H groups which favor non-radiative decay. These groups are present in all complexes; nevertheless, they have a relevant spin density only in **Gd3**. The spin population analysis on NaL models, in which Na^+^ is coordinated to a deprotonated ligand, mimicking the coordinative environment of the complex, confirms the outcomes on the free ligands.

## Abstract 2

A series of Gd^3+^ complexes (**Gd1**−**Gd3**) were studied by combining TR-EPR spectroscopy and DFT calculations to rationalize the deviant spectroscopic behavior of the bulkiest complex (**Gd3**). The combination of ZFS calculations and the spin density delocalization analysis ascribed the larger red shift to the higher degree of delocalization of the wavefunction and the low intensity of the phosphorescence band to the presence of C−H groups with relevant spin density that favor non-radiative decay.

## Introduction

Excited triplet states of chromophore units play an important role in several photophysical and reactive phenomena. Among processes involving them, triplets are of paramount importance in the so-called antenna effect for the sensitization of lanthanide (Ln) ion emission,^1^ as the energy gap between triplet and Ln^3+^ emitter levels is one of the key factors ruling the emission properties.^1^ For instance, lanthanide luminescence-based thermometric features are tightly bound to the triplet state energy, in particular when the back-energy transfer is considered.^2−7^ Besides its energy, the design of novel luminescent systems with tailored properties requires a detailed knowledge of the triplet spin distribution over the molecular skeleton. Indeed, energy transfer pathways are sometimes directly influenced by the specific spatial distribution of the spin density in the sensitizer ligand and by the triplet energy.^8−10^ Moreover, the delocalization of the triplet state spin density can be related to the phosphorescence quantum yield, whose control is crucial in technological applications such as organic light-emitting diodes.^11^

To investigate the triplet formation mechanism, its population, the spin density distribution, time-resolved electron paramagnetic resonance (TR-EPR) spectroscopy, and quantum mechanical modeling have been herein combined. In general, the TR-EPR technique can be used to monitor the evolution of short-lived spin states induced by light excitation^12,13^ and can be applied to triplet,^14^ quartet, and quintet states,^15,16^ spin correlated radical pairs,^17^ and charge-separated states.^18,19^ More specifically, the triplet state TR-EPR spectroscopy provides information about (i) the triplet formation mechanisms from the sub-level populations, (ii) the delocalization and the symmetry of the triplet wavefunction through the zero-field splitting (ZFS) parameters, and (iii) the orientation of the transition dipole moment from magneto-photo selection effects.^20^ Conversely, triplet formation and decay kinetics are not straightforwardly obtained from TR-EPR spectroscopy, being often overshadowed by the faster spin-relaxation.^21^ It is well known that density functional theory (DFT) calculations are suitable for estimating EPR parameters such as the g-tensor.^22^ However, the evaluation of ZFS parameters (*D* and *E*) has proven to be much more challenging. As a matter of fact, the spin contamination has a deep impact on the calculation accuracy, and spin-unrestricted DFT calculations are therefore advised against. The restricted open-shell (RO) approach does not suffer from spin contamination and, even though the wavefunction description might not be as accurate as with the unrestricted formalism, the resulting ZFS parameters are usually in better agreement with the experiment.^23,24^ Furthermore, only spin–spin coupling needs to be taken into account for organic triplets as the spin–orbit contribution is negligible for these systems.^23,24^ Before going on, it has to be remarked that DFT can reproduce trends in *D* and *E* parameters for a series of homologue molecules, but their absolute values are usually underestimated relative to the experimental ones.^23,24^ Multireference methods such as complete active space self-consistent field (CASSCF) are a possible alternative to DFT, but they become impractical as the molecular size, and consequently the active space size, increases. Moreover, CASSCF and DFT calculations provide quite similar results on a wide variety of organic systems.^23^

**Figure 1:**
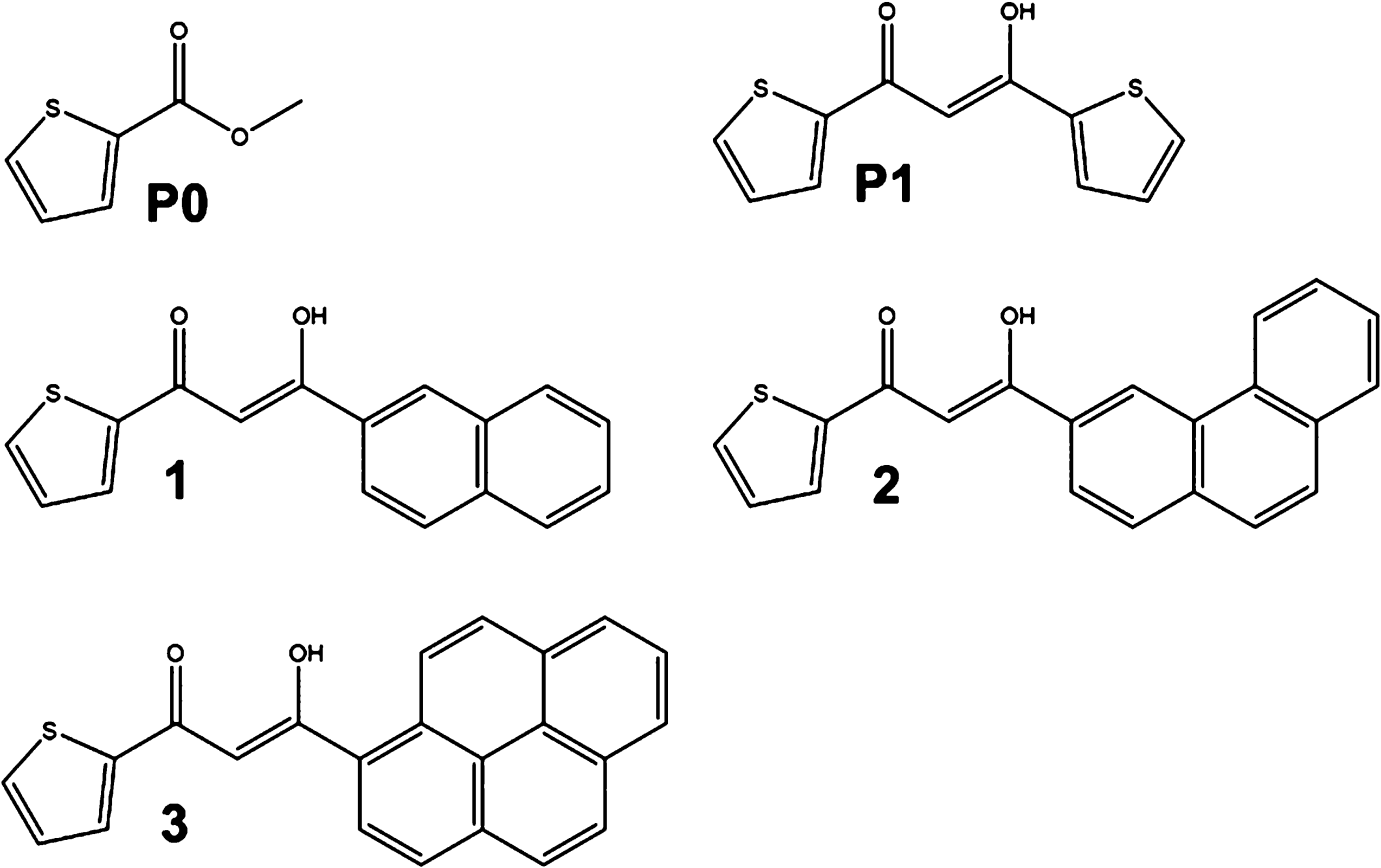
Chemical structures for precursors **P0** and **P1** bearing one and two thienyl rings, respectively, and ligands **1**, **2**, and **3** containing a thienyl ring and a PAH substituent of increasing size (naphthyl, phenanthryl, and pyrenyl).

In this work, a series of Gd^3+^ complexes with the general formula GdL_3_(EtOH)_2_, where L is a β-diketone ligand with polycyclic aromatic hydrocarbon (PAH) substituents of increasing size (**1**–**3**, see Figure 1), have been investigated along with two precursors (**P0** and **P1**, see Figure 1) bearing one and two thienyl rings, respectively, which were considered for assessing the contribution of the thienyl group to the triplet properties.

We started from the observation of the anomalous phosphorescence emission of the bulkiest complex (**Gd3**) compared to **Gd1** and **Gd2**. Indeed, the **Gd3** phosphorescence band is barely observed only at 80 K and red-shifted by ∼200 nm from the most intense fluorescence band. Such a red shift decreases to ∼100 nm in **Gd1** and **Gd2**, whose phosphorescence spectra are clearly visible also at room temperature (RT). Since the origin of the anomalous spectroscopic behavior of **3**/**Gd3** compared to the other compounds might be due to the nature of the triplet states, TR-EPR spectroscopy and DFT calculations have been exploited in an integrated fashion to look into this matter.

## Results and Discussion

**Figure 2:**
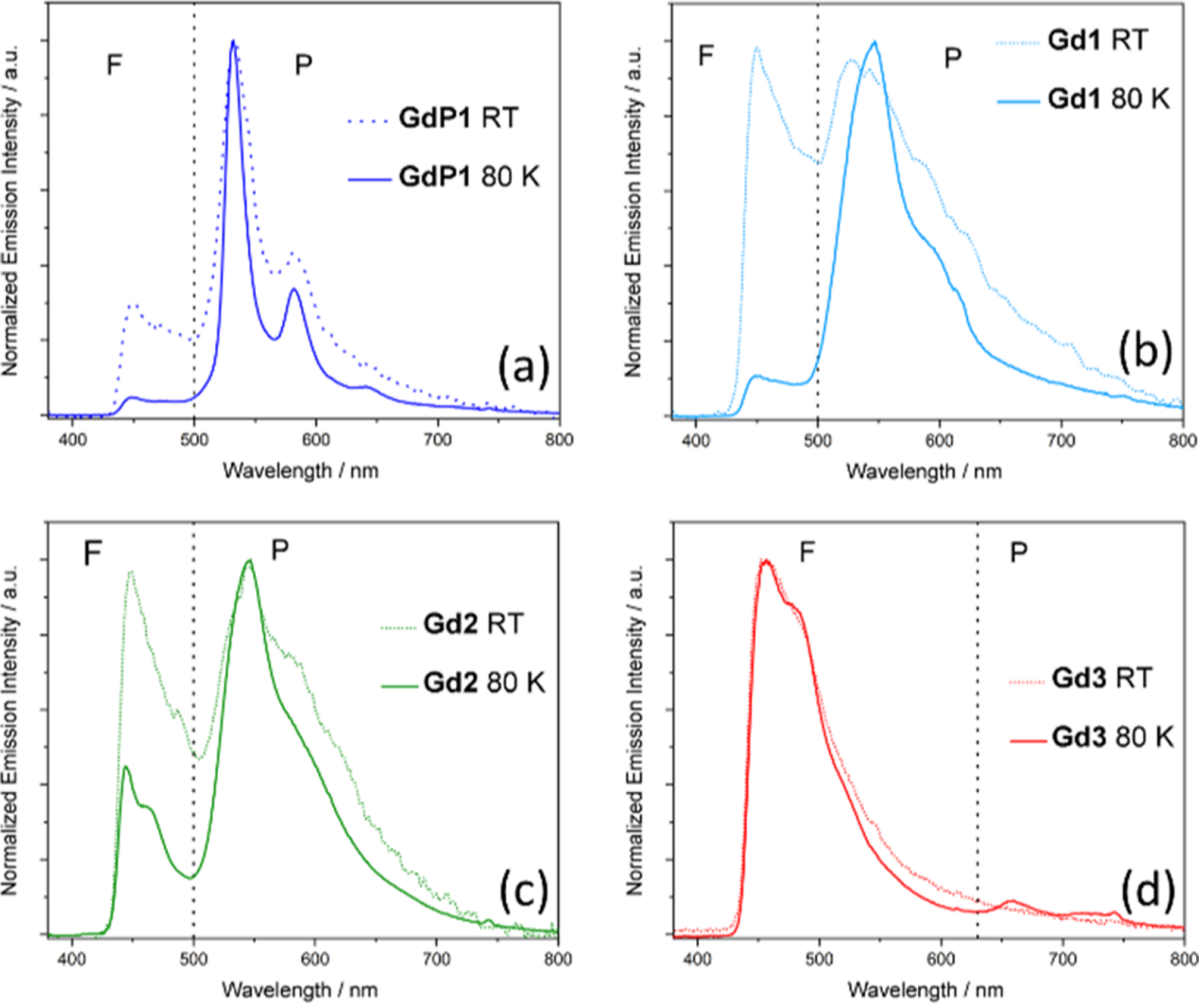
Emission spectra of (a) **GdP1** and (b–d) **Gd1**–**Gd3** complexes at RT and at 80 K. Vertical dashed lines are a guide to the eye to better visualize the region in which the most intense fluorescence (F) and phosphorescence (P) bands are located.

Structural, vibrational, and electronic properties of ligands **1**–**3** have been recently investigated by combining DFT-based methods with X-ray crystallographic data and UV–Vis absorption spectra.^2^ In particular, the analysis of X-ray structures revealed, in agreement with DFT outcomes, the presence of different rotational isomers for the ligands. Triplet energies^2^ were theoretically estimated and the corresponding results compared with the phosphorescence spectra of Gd^3+^ complexes. Further investigations on the emission spectra of **GdP1** and **Gd1**–**Gd3** complexes reveal relevant differences through the series (from **GdP1** to **Gd1**–**Gd3**). Indeed, both fluorescence and phosphorescence bands are present at RT for **GdP1**, **Gd1**, and **Gd2** (Figure 2). The polystyrene films in which the complexes were embedded provided a sufficiently rigid matrix to hamper vibrational motion, thus allowing the observation of phosphorescence bands even at RT. Cooling the sample down to 80 K strongly modifies the relative intensity of fluorescence and phosphorescence bands, with the latter becoming the dominant contribution in the photoluminescence spectra of **GdP1**, **Gd1**, and **Gd2**. Conversely, the **Gd3** 80 K phosphorescence emission is barely observable at wavelengths longer than 630 nm and it appears red-shifted by approximately 200 nm from the intense fluorescence band. For the other complexes, this shift is approximately 100 nm. This evidence cannot be explained by simply considering the emission data and the calculations of the energy of the ground (singlet) and triplet states.^2^ Insights into such a peculiar behavior may be gained by combining TR-EPR spectroscopy with DFT calculations.

**Figure 3:**
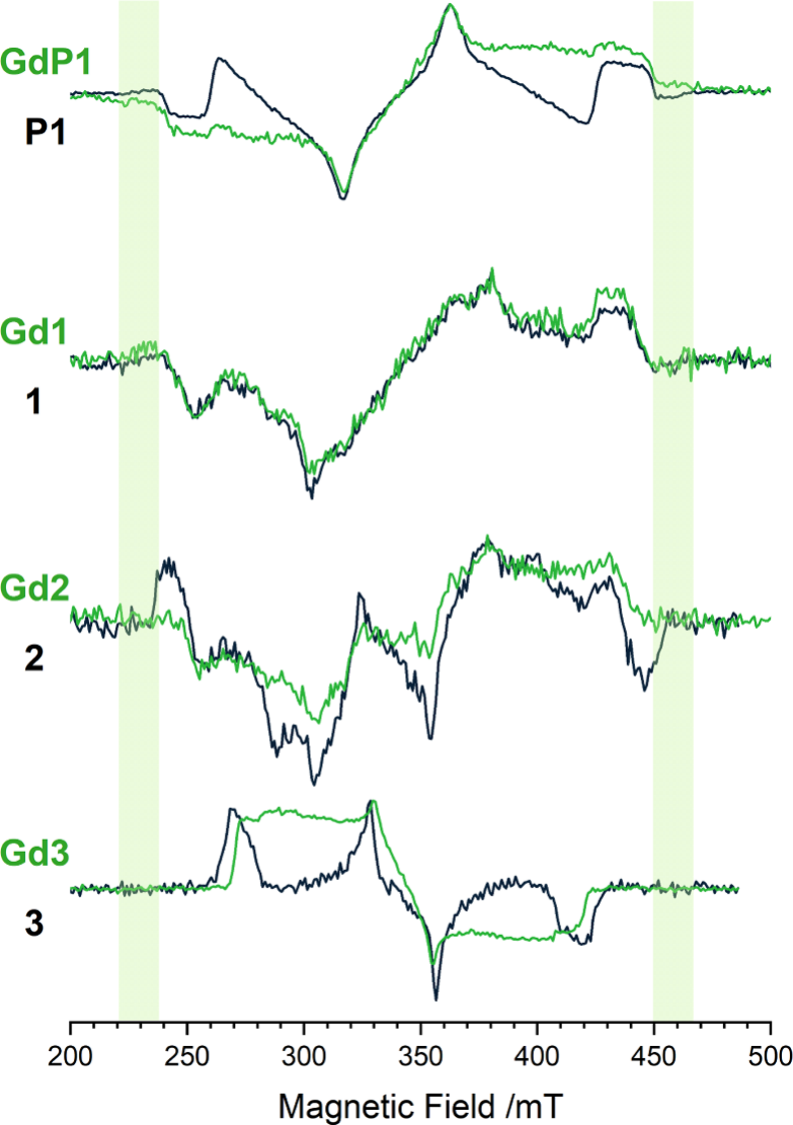
TR-EPR spectra (λ_exc_ = 355 nm) of the precursor **P1** and ligands **1**–**3** (black lines) and Gd^3+^ complexes **GdP1**, **Gd1**–**Gd3** (green lines) in frozen toluene solution at the X-band (ν = 9.705 GHz), *T* = 80 K. The green side-bands correspond to the maximum width of the spectra of **GdP1**, **Gd1**, and **Gd2** (equal to 2|*D*| × *h*/*g*μ_B_) and highlight the progressive narrowing of the EPR spectra.

**Figure 4:**
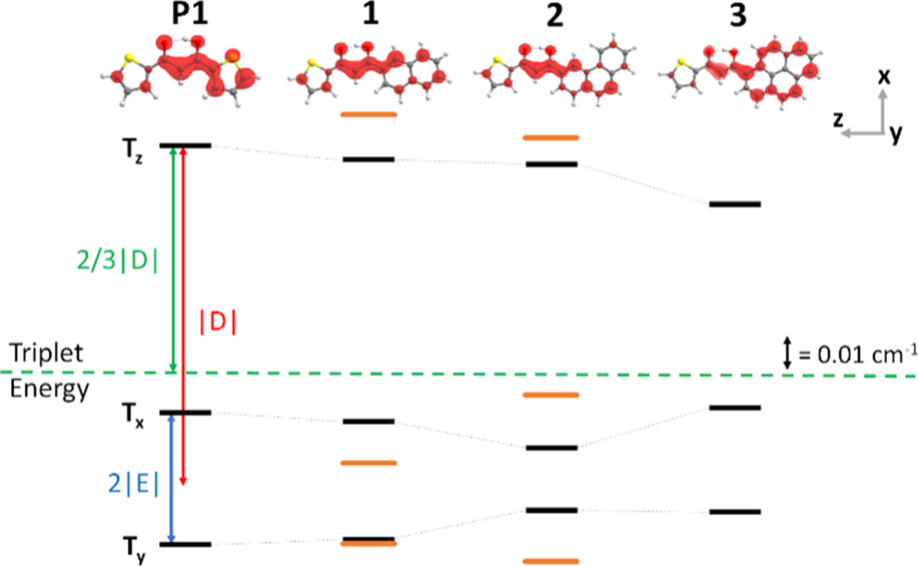
Top, the spin delocalization of the triplet state in the main conformer of the precursor **P1** and ligands **1**–**3**. The axes describe the orientation of the main ZFS reference frame relative to the molecular skeleton for all the molecules (see Figure S4 of the Supporting Information for details). Gray, yellow, red, and white spheres are C, S, O, and H atoms, respectively. Bottom, energies of the triplet sublevels (T_*x*_, T_*y*_, and T_*z*_) relative to the triplet energy (green dashed line) based on the |*D*| and |*E*| experimental values for the precursor **P1** and all ligands (in cm^–1^). The scheme has been drawn using the signs of the ZFS parameters obtained from the calculations (*D* < 0; *E* < 0). Black and orange bars represent the different species where the former is the species with the highest spectral percentage.

TR-EPR spectra of **P1, 1**–**3** and **GdP1, Gd1**–**Gd3** in frozen solutions (80 K) are reported in Figure 3, while simulated TR-EPR spectra for ligands and complexes are displayed in Figures S1 and S2 of the Supporting Information. As far as the simulation parameters are concerned, they are collected in Table 1. Spectra simulations allowed us to obtain: (i) ZFS parameters of the triplet states; (ii) populations of the triplet sublevels (spin polarizations); and (iii) the relative amount of different triplet spectral contributions when more than one is present. Only relative spectral contributions can be evaluated since the absolute intensity of a TR-EPR spectrum depends on spin polarization, on the extinction coefficient of different species at the excitation wavelength (see Figure S3 in the Supporting Information), and on several hard to control experimental parameters. Moreover, absolute values of ZFS parameters are reported in Table 1 because the direct experimental determination of the *D* and *E* signs was beyond the scope of this work and far from trivial.^25^ Nevertheless, as the software package employed for simulations needs the sign for the ZFS parameters, a negative sign for *D* and *E* has been adopted based on the results of DFT calculations (*vide infra*); thus, the three triplet sublevels in order of increasing energy are T_*y*_, T_*x*_, and T_*z*_ (see also Figure 4).

**Table 1:** Triplet Parameters Obtained from the Simulations of the TR-EPR Spectra when Two Species are Present; Each Row Reports Two Sets of Parameters^a^

	|*D*|	|*E*|	|*E/D*|	P_*x*_/P_*y*_*/*P_*z*_	%
**P0**	0.111	0.030	0.270	0.80:0.00:0.20	100
**P1**	0.098	0.019	0.194	0.00:0.49:0.51	>95
	0.111	n.d.	n.d.	n.d.	n.d.
**GdP1**	0.098	0.019	0.194	0.00:0.39:0.61	>95
	0.111	n.d.	n.d.	n.d.	n.d.
**1** & **Gd1**	0.092	0.017	0.185	0.00:0.00:1.00	63
	0.111	0.013	0.117	1.00:0.00:0.00	37
**2**	0.090	0.008	0.089	0.07:0.00:0.93	62
	0.100	0.024	0.240	0.93:0.07:0.00	38
**Gd2**	0.090	0.008	0.089	0.00:0.02:0.98	73
	0.100	0.024	0.240	0.90:0.00:0.10	27
**3**	0.074	0.016	0.216	0.79:0.21:0.00	100
**Gd3**	0.070	0.016	0.229	0.49:0.51:0.00	100

a Absolute values of the ZFS parameters |*D*| and |*E*| (cm^–1^); |*E/D*| ratio; triplet sublevel population (P_*x*_, P_*y*_, and P_*z*_); relative amount of each spectral component (%). The *g* tensor for all is *g*_xx_ = 2.006, *g*_yy_ = *g*_zz_ = 2.009. n.d. = not determined.

The lineshape analysis of the **GdP1** and **Gd1**–**Gd3** TR-EPR spectra suggests that triplet states are populated *via* intersystem crossing (ISC) from the first excited singlet state rather than singlet fission or recombination of a radical pair as these would both lead to drastically different polarizations (and thus lineshapes).^13,25^ The **GdP1** TR-EPR spectrum is dominated by a single triplet species (only the wings of a second larger species are visible as highlighted by the green bands in Figure 3), while the **Gd1** and **Gd2** ones are consistent with the presence of two triplet species (the simulations of the individual species are reported in Figure S2 of the Supporting Information). The **Gd3** spectrum is characterized by the presence of a single triplet state. The presence of multiple triplet species for **Gd1** and **Gd2** cannot be attributed to the contributions from higher excited triplet states (T_2_, T_3_, ...) since these would relax to T_1_ too quickly to be detected by TR-EPR. They may be ascribed to different rotamers—whose presence was previously observed from the X-ray structures^2^—with a different delocalization of the triplet wavefunction and thus different ZFS parameters.

To disentangle the role of the Gd^3+^ ion on the observed triplet states properties, TR-EPR spectra of the free ligand have also been recorded. The **P1/GdP1**, **1/Gd1**, and **2/Gd2** spectra and parameters are very similar, thus indicating a marginal role played by the Ln^3+^ ion. The largest variations involve the triplet sublevel population ratios, thus suggesting that the metal ion modifies only the triplet sublevel population, that is, it affects the ISC process. Since the presence of Gd^3+^ does not perturb the ZFS parameters of the triplet state and only the relative amounts of the rotamers are possibly affected, their conformation (and thus spin distribution) remains unchanged in the complexes. On the contrary, when the **3**/**Gd3** pair is considered, markedly different spectra and parameters are observed (*vide infra*), suggesting a structural conformational change induced by the complex formation.

Energies of the triplet sublevels (T_*x*_, T_*y*_, and T_*z*_) relative to the triplet state energy (dashed line) are displayed for **P1**, **1**–**3** in Figure 4, where black bars refer to the main species, while orange bars refer to the minority species. Both Figure 4 and Table 1 highlight that |*D*| values decrease upon increasing the PAH size (**P1**, **1** → **3**), while a clear trend is not evident for *E*. As such, the decrease of *D* along the series accounts for a progressively broader delocalization of the triplet wavefunction over the molecular skeleton (see the spin densities for the main species on the top of Figure 4). The trend in the ZFS parameters can be analyzed in terms of the *E/D* ratio (see Table 1), which indicates the symmetry of the spin distribution, from purely axial (*E/D* = 0) to fully rhombic (*E/D* = 1/3). The main spectral species show a clear reduction of the *E/D* ratio moving along the series, indicating a progressively more axial distribution, from **P0**/**P1** to **1**/**2** (and the corresponding complexes), but again the **3**/**Gd3** pair deviates from this trend. Further information about the roles played by the thienyl and PAH fragments has been gained by recording and simulating the TR-EPR spectra of **P0**, a precursor only bearing the thienyl moiety. Experimental and simulated spectra of **P0** are reported in Figure S1 of the Supporting Information, while the relative ZFS parameters are reported in Table 1. The triplet species of **1** is narrower (|*D*| = 0.098 cm^–1^) than that observed for **P0**, thus indicating a larger delocalization; however, |*D*| for **P1** is not greatly reduced compared to **P0** as it could be expected if the triplet state were fully delocalized from one thienyl ring to the other in **P1**. Such evidence necessarily implies that the two thienyl rings of **P1** are not equivalent. In this regard, it is worth mentioning that the reduction of the ZFS in conjugated structures with progressively increasing repeating units depends not only on the extent of the delocalization but also on the ZFS axes’ direction.^26^ Moreover, |*D*| values pertaining to the main triplet species in **1** (|*D*| = 0.092 cm^–1^) and **2** (|*D*| = 0.090 cm^–1^) and to the only species in **3** (|*D*| = 0.073 cm^–1^) are similar but slightly smaller than those of the corresponding PAH^27^ (naphthalene, |*D*| = 0.101 cm^–1^, for **1**; phenanthrene, |*D*| = 0.105 cm^–1^, for **2**; pyrene, |*D*| = 0.086 cm^–1^, for **3**). Such a result seems to indicate that in the main species of the asymmetric ligands, the wavefunction is delocalized on both the β-diketone part and the PAH moiety without any involvement of the thienyl ring. Note that a triplet state delocalized over most of the ligand molecules represents a prolate spin density distribution, which implies a negative *D* parameter as found by the DFT calculations (*vide infra*).^25^

TR-EPR spectra and their analysis also provide useful information to rationalize the **GdP1** and **Gd1**–**Gd3** fluorescence and phosphorescence measurements. In this regard, (i) the **3**/**Gd3** spectra confirm that the corresponding triplet levels are effectively populated; hence, the low phosphorescence cannot be associated with the difficulty of populating the triplet state; (ii) the lowest |*D*| values in **3**/**Gd3** clearly indicate the largest delocalization among the ligands. As a whole, TR-EPR spectra of **3**/**Gd3** do not show any evidence that may justify the very weak phosphorescence emission, even in a rigid matrix, at 80 K.

Taking these results as a starting point, we performed a series of DFT numerical experiments to investigate the triplet states through the evaluation of ZFS parameters and to gain insights into the spin delocalization. The latter aspect is crucial because the delocalization of the triplet state spin density provides information about the possible regions of the molecules where the spin–orbit coupling may occur.^11^ The large similarity observed between the ZFS parameters of the complexes and those of the free ligands supports the commonly accepted assumption that in Ln^3+^ antenna complexes, the excitation is localized on the ligands and the emission on the lanthanide. This implies that the central metal and the ligands are mostly independent, and electronic properties are substantially unaffected upon moving from the isolated fragments to the complex.^2,28,29^ Thus, the smaller size of the free ligand compared to that of the corresponding complex allows the estimation of ligand ZFS parameters through more accurate calculations, and the results can be then transferred to their Gd^3+^ complexes.

**Figure 5:**
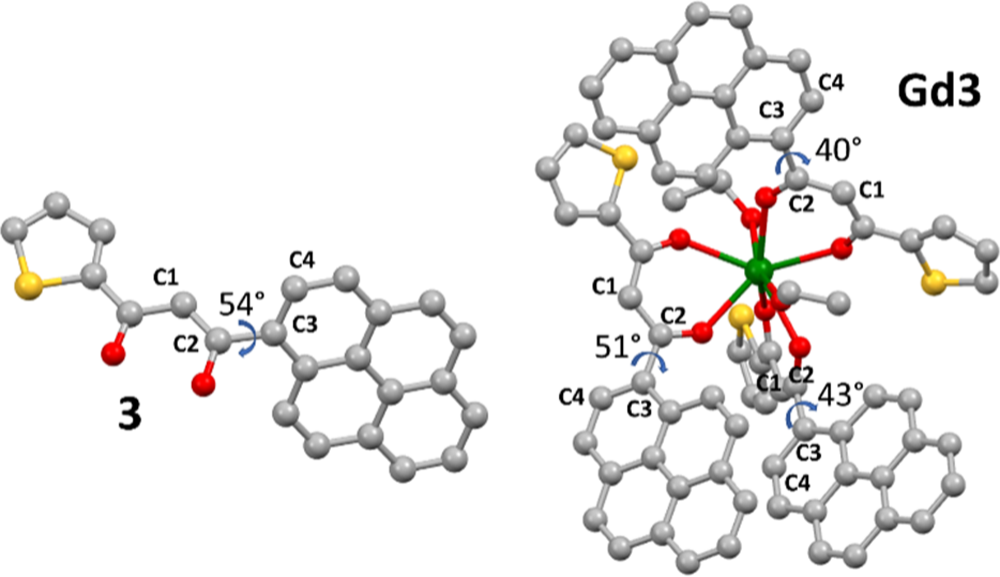
Comparison between the pyrenyl group orientations in **3** and **Gd3**. Gray, yellow, red, and green spheres are C, S, O, and La atoms, respectively. H atoms are omitted for clarity. Dihedral angles are given in degrees.

Before discussing the results of the ZFS calculations pertaining to ligands, it is crucial to underline the similarities and the differences of the optimized structures for the **P1**, **1**–**3**/**GdP1**, and **Gd1**–**Gd3** pairs. Experimental crystal structures of **P1** and **1**–**3** are reported in the literature.^6^ The comparison between ground-state optimized geometries for **P1**, **1**–**3** and **GdP1**, **Gd1**–**Gd3** reveals that, in **P1**, **1** and **2** and **GdP1**, **Gd1**, and **Gd2**, the PAH groups have almost the same orientation. Indeed, the **GdP1**, **Gd1**, and **Gd2** average dihedral angles Φ (defined as C1–C2–C3–C4, Figure 5) are 4, 20, and 27°, respectively, and are very close to the values of **P1**, **1**, and **2** (Φ = 0, 20, and 23°, respectively). At variance to that, the bulky pyrenyl group in **Gd3** and **3** is characterized by significantly different twist angles (average Φ = 45° in the former, Φ = 54° in the latter) to favor the coordination of three ligands to the Gd^3+^ (see Figure 5). Different |*D*| values in **3** and **Gd3** are then tentatively ascribed to the diverse Φ angles upon moving from **3** and **Gd3**.

To evaluate ZFS parameters, triplet geometries for **P0**/**P1** and **1**–**3** have been optimized. Experimental crystal structures of **P1** and **1**–**3** are consistent with the presence of multiple rotamers differing for the relative orientation of the aromatic rings.^2^ Triplet geometries needed for ZFS parameters have therefore been optimized for all four possible rotamers, herein labeled A, B, C, and D (see Figure S5 of the Supporting Information). B and C rotamers may be obtained by flipping either the thienyl moiety (B) or the PAH fragment (C) of the predominant species A. Rotamer D is generated by flipping both the thienyl group and the PAH fragment. Relative energies of optimized structures are systematically within 2 kcal/mol of the most stable rotamer. The relatively low energy barriers for the rotation of the aromatic fragment around the bond with the diketone moiety suggest a substantially free ring rotation in solution and thus the presence of all possible rotamer configurations.^30^ The detailed description of the calculations for rotational barriers and their values have been reported in a previous work.^2^ Experimental and theoretical |*D*| and |*E*| triplet values for all possible rotamers of **P0**/**P1** and **1**–**3** are reported in Table 2.

**Table 2:** ZFS Parameters *D* and *E*/*D* for All Rotamers of **P0**, **P1**, and **1–3**^a^

		RO-BP86		experimental	
		*D* (%)	*E/D*	|*D*| (%)	|*E/D*|
**P0**	A/B	–0.070(21)/–0.071(79)	0.329/0.324	0.111	0.270
**P1**	A/B	–0.067(42)/–0.068(27)	0.209/0.206	0.098	0.194
	C^b^/D	–0.068(27)/–0.080(4)	0.206/0.125		
**1**	A/B	–0.071(5)/–0.081(3)	0.085/0.062	0.092(63)	0.185
	C/D	–0.048(56)/–0.048(36)	0.292/0.208	0.111(37)	0.117
**2**	A/B	–0.070(20)/–0.070(13)	0.100/0.100	0.090(62)	0.089
	C/D	–0.050(41)/–0.049(26)	0.280/0.286	0.100(38)	0.240
**3**	A/B	–0.039(20)/–0.039(17)	0.128/0.179	0.074	0.216
	C/D	–0.031(36)/–0.031(27)	–0.226/0.226		

a *D* parameter is given in cm^–1^. Calculated RO-BP86% are taken on the optimized triplet-state geometries considering the energy difference between the rotamers (A/B/C/D) according to a Boltzmann population at 298.15 K and are reported in parentheses in the *D* column.

b For **P1**, with two thienyl rings, the B and C rotamers are equal.

Two different functionals, GGA-(BP86) (in Table 2) and hybrid (B3LYP), have been tested and the results are very similar (Table S2 of the Supporting Information). In agreement with the literature,^23^ theoretical calculations of *D* and *E* underestimate the experimental values by ∼30–40%; nevertheless, the |*D*| trend through the series, similar values for **P1**, **1**, and **2** and a much lower value for **3**, is satisfactorily reproduced. The experimental trend of the *E/D* ratio is not well reproduced moving along the series, but interestingly it shows that A/B conformers, in general, have a more axial distribution, whereas C/D conformers have a more rhombic distribution. A similar behavior has been observed experimentally when two species are present, that is, the main species is more axial while the minor species is more rhombic. It has already been mentioned that TR-EPR spectra of **1** and **2** suggest the presence of two species, while those of **P0**/**P1** and **3** are consistent with the occurrence of a single species. This perfectly matches the RO-BP86 results (see Table 2): in **P0**, **P1**, and **3**, *D* and *E* values corresponding to the relevant species of **P0**/**P1** and **3** are very close; meanwhile, for **1** and **2**, the ZFS parameters of A/B rotamers significantly differ from those of the C/D ones, consistent with the presence of two magnetically active species. The hypothesis that different rotamers with different spin delocalizations^31^ and ZFS parameters contribute to the TR-EPR spectra is then fully supported by DFT calculations.

**Figure 6:**
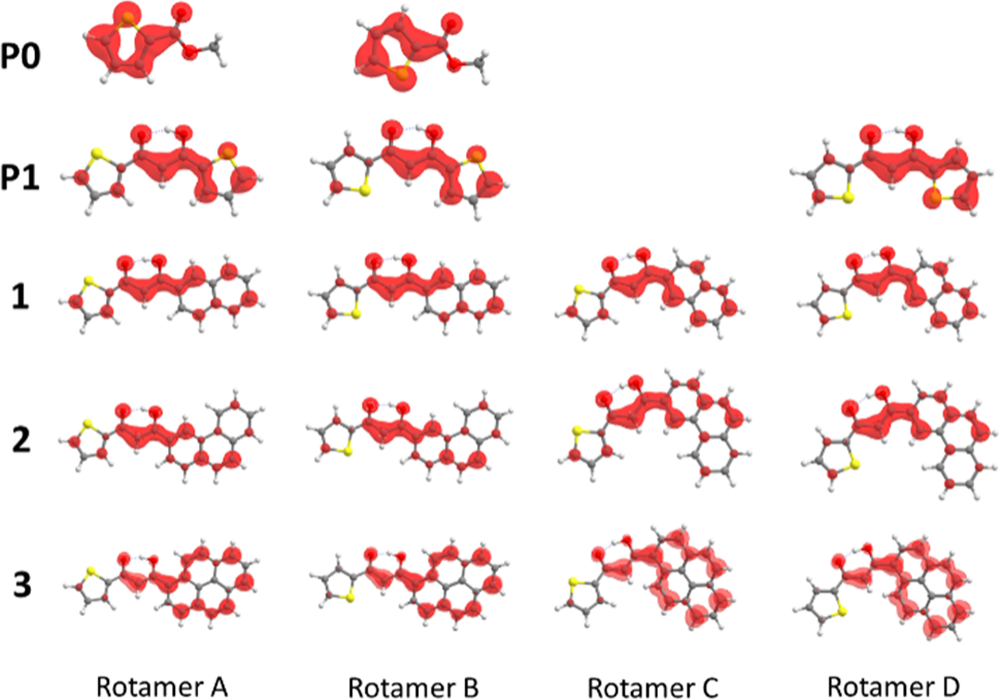
Spin densities of all rotamers for **P0**, **P1**, and **1**–**3** calculated at the RO-BP86 level. The displayed isosurfaces correspond to 0.003 × 10^1/2^ × Å^–3/2^ values. Gray, white, yellow, and red spheres are C, H, S, and O atoms, respectively. For **P1** with two thienyl rings, the B and C rotamers are equal.

The different *D* and *E/D* parameters in A/B and C/D rotamers imply different spin densities (whose 3D plot are displayed in Figure 6). More specifically, the spin density analysis of the **P0**/**P1**, **1**–**3** rotamer A reveals that: (i) the spin density on the thienyl moiety decreases upon increasing the PAH size; (ii) the diketone fragment of all but one ligand (**3**) is always populated; and (iii) the spin density values are only slightly affected by the PAH size. Despite the fact that the |*D*| trend is properly reproduced for **P1**, **1**–**3**, we cannot be silent about a minor discrepancy between experiment (|*D*(**P1**)| > |*D*(**1**)|) and theory (|*D*(**P1**)| ≈ |*D*(**1**)|). Indeed, the spin density on the thienyl ring (enol side) in **P1** appears too high compared to the other ring.

Spin density differences can be qualitatively evaluated by considering Lüwdin^32^ or Mulliken^33^ spin populations. The spin density is a function of the three-dimensional space and the spin populations simply correspond to the spin density breakdowns onto the atoms, making it possible to assign percentage values (see Table S3 of the Supporting Information) to different fragments. The spin population is mainly localized on the diketone moiety in **P1**, **1**, and **2**, while a pronounced spin density shift on the PAH fragment takes place in **3**. More specifically, the spin density % localization on the diketone decreases from ∼50 to ∼20% upon moving from **P1**, **1**, and **2** to **3** while on the PAH fragment, it increases from ∼40 to >70%. These outcomes are consistent with both the larger phosphorescence red shift observed for **Gd3** and its lower phosphorescence yield. As far as the former point is concerned, a higher degree of delocalization in the wavefunction with respect to the other ligands (see Figure 6) results in a higher stabilization for the corresponding triplet state and therefore a larger energy gap between the excited S_1_ and T_1_ states, that is, a larger shift between the fluorescence and phosphorescence bands.^34,35^ Indeed, while paired electrons mostly repel each other *via* Coulomb interaction, the exchange term, which characterizes electrons with the same spin, is less pronounced as the delocalization of the wavefunction increases, therefore stabilizing the corresponding triplet states for a relatively more delocalized triplet (pyrene) compared to a more localized one (diketone). Moving to the latter point, the low intensity of the phosphorescence band can be associated with relevant non-radiative triplet decay pathways. The efficiency of the non-radiative decay processes is tied to two quantities: the energy gap between the two electronic states of interest (in our case T_1_ and S_0_) and the presence of high-energy oscillators. This relation^36^ has been successfully applied to several systems to explain the phosphorescence trend of a series of conjugated polymers and monomers^37^ or the luminescence efficiencies of transition-metal complexes.^38^

High-energy oscillators such as the C–H stretching mode have already been proven to cause lower phosphorescence yields and excited state lifetimes in similar organic compounds.^39,40^ However, our DFT outcomes demonstrate that the mere presence of a high-energy oscillator is not enough to explain this behavior. Indeed, the **P1** precursor and all ligands **1**–**3** feature C–H groups able to contribute to non-radiative decay, but they have different phosphorescence yields. This is because in **P1**, **1**, **2**/**GdP1**, **Gd1**, and **Gd2** the spin density is primarily localized on the diketone fragment, where only a single C–H oscillator is present (Figure 6 and Table S3 of the Supporting Information), whereas in **3**/**Gd3**, the triplet is localized on the C–H oscillator of the pyrenyl moiety. In our case, therefore, the combination of a more stable triplet state in **3**/**Gd3** and the presence of a high number of C–H oscillators in the pyrenyl fragment bearing the spin density contributes to significantly more efficient non-radiative decay processes compared to **P1**, **1**, **2**/**GdP1**, **Gd1**, and **Gd2**.

Results so far obtained provide information about the origin of the anomalous behavior of the **Gd3** phosphorescence spectra, both in terms of intensity and red shift. Aimed to better model the Gd^3+^ coordinative environment and to obtain also a quantitative agreement with experimental data, the deprotonated ligand (L^–^) has been coordinated to a Na^+^ ion (see Figure S7 of the Supporting Information where the **Na1** model is displayed) for a further series of numerical experiments. The triplet geometries of the **NaP1** and **Na1**–**Na3** models have been optimized for all rotamers. *D* and *E/D* values for rotamer A are reported in Table 3, while values for all the rotamers are collected in Table S4 of the Supporting Information.

**Table 3:** Calculated ZFS Parameters *D* and *E/D* for **P1**, **1–3**, for **NaP1**, **Na1**–**Na3** Models (Rotamer A) and **GdP1**, **Gd1**–**Gd3** in the Lowest Energy Triplet State^a^

	*D* (*E/D*)			|*D*| (|*E/D*|)	
	calculated			experimental	
	L	NaL model	Gd^3+^ complex	L	Gd^3+^ complex
**P1**	–0.067 (0.209)	–0.078 (0.137)	–0.072 (0.111)	0.098 (0.194)	0.098 (0.194)
**1**	–0.071 (0.085)	–0.080 (0.114)	–0.072 (0.107)	0.092 (0.185)	0.092 (0.185)
**2**	–0.070 (0.100)	–0.072 (0.097)	–0.073 (0.068)	0.090 (0.089)	0.090 (0.089)
**3**	–0.039 (0.128)	–0.040 (0.118)	–0.036 (0.117)	0.074 (0.216)	0.070 (0.229)

a *D* parameter is given in cm^–1^. Absolute experimental values for ligand and Gd^3+^ complexes are reported for comparison. Level of theory: RO-BP86.

The inspection of Table 3 highlights a better agreement between experiment and theory, particularly evident for the smallest models (**NaP1** and **Na1**), suggesting that the constraints induced by the sodium coordination are sufficient to improve the agreement. The poorer enhancement characterizing bulkier models, especially **Na3**, is probably due to the larger geometrical variations between the isolated and the coordinated ligand in the whole complex, which is not captured by the simplified model. This assumption was demonstrated for singlet ground-state Gd^3+^ complex geometries, where the dihedral angles are compared (see above). The comparison between the rotamer spin densities for **P1**, **1**–**3** (Figure 6) and **NaP1**, **Na1**–**Na3** optimized triplet states (Figure S8 of the Supporting Information) reveals that the **NaP1** and **Na1** spin density is more localized on the diketone moiety with respect to the free ligand one. Negligible variations are instead found for larger models (**Na2** and **Na3**). Spin population analysis was also performed for the **NaP1** and **Na1**–**Na3** models and the outcomes are very similar to the ligand ones. Not only the trend is the same, but the percentage values themselves are close (see Table S3 of the Supporting Information).

Calculations of ZFS parameters have been extended to the deprotonated ligands (**L**^–^, rotamer A) as well in order to evaluate the effect of the counterion (H^+^ or Na^+^). *D* values of L and L^–^ clearly indicate a better agreement for the former species (see Table S5 of the Supporting Information). As such, it is noteworthy that the deprotonated species **3** has the highest |*D*| value, while, according to the experiments, the protonated form **3** has the lowest |*D*| value. Analogous considerations hold for spin densities (see Figure S9 of the Supporting Information). The presence/absence of the proton slightly affects the spin density distribution of **P1** and **1**, whereas it strongly influences that of **2** and **3**. Similar trends can be drawn by comparing L^–^ and NaL. The H^+^/Na^+^ coordination to the O atom is then crucial for reproducing the experimental trend.

The last computational step concerned the evaluation of **GdP1** and **Gd1**–**Gd3** ZFS parameters. Optimized geometries of the lowest energy triplet states have been obtained and the corresponding ZFS relative parameters are collected in Table 3. The comparison between the calculated and the experimental ZFS values of **GdP1**, **Gd1**–**Gd3**/**NaP1**, and **Na1**–**Na3** reveals that the best qualitative and quantitative agreement is obtained for **NaP1** and **Na1**–**Na3** models. This suggests that calculations on the Gd^3+^ complexes are unnecessary, and a simpler model, able to correctly mimic the ligand coordination to a central ion, is more than sufficient.

## Conclusions

A series of β-diketone ligands featuring a thienyl ring and a PAH fragment of varying size and their Gd^3+^ complexes has been investigated to rationalize the different behavior of the emission spectra for the largest system (**Gd3**). Indeed, its phosphorescence band is only barely observed at 80 K and a large red shift with respect to the fluorescence band is revealed. To gain information on the triplet states and to explain the spectral trend, all ligands and complexes have been investigated both experimentally and theoretically by combining TR-EPR spectroscopy and DFT calculations. TR-EPR spectra of the Gd^3+^ tris-β-diketonate complexes for **P1**, **1**, and **2** are similar to those of the free species, ultimately stating that the triplet nature is unchanged upon complexation. The different behavior of the **3**/**Gd3** pair is attributed to a different twist of the pyrenyl group in the free ligand compared to the coordinated one, as highlighted by DFT outcomes. Moreover, TR-EPR spectra found that the triplet populations in **3** and **Gd3** are significant; hence, the low phosphorescence intensities observed are not due to the low triplet yield. The smallest |*D*| values of **3** and **Gd3** found by TR-EPR analysis suggested a broader electron spin density delocalization on the ligands.

Starting from these results, DFT calculations for estimating the ZFS parameters have been performed on (i) free ligands; (ii) a model with the deprotonated ligands coordinated to a Na^+^ ion; (iii) the deprotonated ligands; and (iv) the Gd^3+^ complexes. Calculated ZFS parameters confirmed the smallest *D* values for **3** and **Gd3** and also a larger delocalization on the PAH moiety. The combination of ZFS calculations, spin density delocalization, and spin population analysis clearly shows the different behavior of **3** and **Gd3** with respect to the other ligands and complexes, which can explain the low intensity of the phosphorescence band and the large red shift of **Gd3**. Indeed, the latter derives from the high degree of delocalization of the wavefunction of **Gd3**. An extended delocalization implies a larger triplet state stabilization and hence a larger energy gap between the excited S_1_ and T_1_ states. The low intensity of the phosphorescence band suggests the presence of very relevant non-radiative triplet decay, which is favored by the lower energy of the T_1_ state and the presence of C–H groups. All Gd^3+^ complexes have a relevant number of C–H groups in the aromatic fragments, but DFT spin density calculations found that only in **Gd3**, the spin density is localized on these groups, hence contributing to the non-radiative decay process. Results concerning the spin density and spin populations analysis on the different fragments of the ligand show that (i) high energy oscillators (i.e., C–H groups) may play a significant role in the non-radiative decay process, but (ii) the mere presence of these groups is not a sufficient condition to rationalize their behavior since they must also carry a relevant spin density. These outcomes could be relevant to drive the design of novel systems in which the non-radiative decay paths from the triplet states can be tuned and controlled.

## Experimental Section

### Synthesis and Characterization

Synthesis and characterization of the precursor **P1** and ligands **1** and **2** (see Figure 1) and corresponding Gd^3+^ complexes (**GdP1**, **Gd1**, and **Gd2**) are reported in ref (6), while those of **3** and **Gd3** are thoroughly described in ref (2). Emission spectra were collected with a Horiba Flurolog 3 spectrofluorometer. **GdP1** and **Gd1**–**Gd3** were embedded in polystyrene thin films and deposited via spin-coating on 10 × 10 mm^2^ fused silica slides.^6^ Temperature was controlled by using a Linkam THMS600 heating/freezing microscope stage coupled with the spectrofluorometer *via* optical fibers. We determined the nature of the emission performing time-gated experiments at 80 K in which a 300 μs delay after the excitation pulse was used to detect slow components (phosphorescence) of the emission spectra. This procedure is commonly employed to isolate the phosphorescence emission of Gd^3+^ complexes and to determine the energy of the triplet levels. We discussed these points in ref (2) where the complete energy level calculation is also reported. A calculation confirmed the nature of the observed transitions. In this work, we used a continuous source for a technical reason. Since the phosphorescence bands of **3** and **Gd3** are faint, we needed high excitation intensity for their detection. The pulsed Xe lamp does not provide enough excitation intensity.

### EPR Spectroscopy

All molecules were dissolved in toluene with a small addition of CH_3_CN and/or CHCl_3_ for solubility; solutions were placed in quartz tubes (i.d. 3 mm), degassed, and sealed under vacuum. The concentration of all samples was approximately 300 μM. TR-EPR experiments were performed at 80 K on a Bruker ELEXSYS E580 spectrometer equipped with an ER 4118X-MD5 dielectric cavity, an Oxford CF935 liquid helium flow cryostat, and an Oxford ITC4 temperature controller. The microwave frequency was measured by a frequency counter, HP5342A. An Nd:YAG laser (Quantel Brilliant) was used for photoexcitation: the laser was equipped with second and third harmonic generators for laser pulses at 355 nm; laser pulses were 5 ns long with an average energy of 5 mJ. TR-EPR experiments were carried out by recording the time evolution of the EPR signal after the laser pulse with a LeCroy LT344 digital oscilloscope. At each magnetic field position, an average of about 1000 transient signals was usually recorded; 300 points on the magnetic field axis were recorded, with a sweep width of 310.0 mT. The microwave power for TR-EPR experiments was set to be low enough (20–25 dB attenuation, i.e. 1.5 mW or less) to be in a low-power regime and avoid Torrey oscillations on the time trace. The time versus field surfaces were processed using a home-written MATLAB program that removes the background signal before the laser pulse (signal vs magnetic field) and the intrinsic response of the cavity to the laser pulse (signal vs time). The TR-EPR spectra shown in the main text were extracted from the surface at 1500 ns from the laser flash, about 100 ns after the maximum in the transient to avoid potential distortions. TR-EPR spectral simulations were performed with EasySpin version 6.0.0—dev34.^41^ The ZFS parameters have been estimated directly from the spectra; the populations and relative amounts of the different spectral components (and, when needed, the anisotropic linewidths) have been obtained by automated fitting using a Levenberg–Marquardt algorithm within the EasySpin package (esfit function). The g and ZFS tensors have been assumed to be collinear. All parameters are reported in Table S1 of the Supporting Information.

### Computational Details

DFT calculations were carried out by using the Orca suite of programs (version 4.2.1).^42^ The hybrid PBE0 functional^43,44^ coupled to an all-electron triple-ζ quality Ahlrichs basis set with one polarization function (def2-TZVP^45^) for all atoms was employed to optimize the molecular structures of singlet (S = 0) ground and excited states and the triplet (S = 1) excited state; for the optimization of the open-shell systems, spin-unrestricted DFT was employed. Coulomb and exchange integrals were approximated by using the Resolution of Identity approximation with the def2/JK auxiliary basis set.^46^ Dispersion corrections were included by adopting Grimme’s DFT-D3 method.^47^ As the lanthanide primarily interacts with the ligands via electrostatic forces and the eventual 4f electrons do not actively take part in the complexation, Gd was substituted with La to obtain a closed-shell system and simplify the SCF convergence in the geometry optimization. The NaL models were obtained by taking the optimized complex geometry and eliminating everything but one ligand and the metal, substituting the lanthanide with a Na^+^ atom, and finally carrying out the optimization on the model system. ZFS parameters were evaluated by using the approaches described in refs (23) and (24) and implemented in the Orca suite. Incidentally, only the spin–spin contribution to the *D* tensor was considered in DFT calculations as spin–orbit effects are negligible for organic systems.^23,24^ As such, the GGA BP86^48,49^ and the hybrid B3LYP^50−52^ functionals in their RO formalism (RO-BP86 and RO-B3LYP) were used together with the def2-TZVP basis set.^45^

## Supporting Information Available

TR-EPR spectra and the relative simulations for all ligand and all complexes; absorption spectra for precursor **P1** and ligands **1**–**3**; ZFS principal axes system for ligands and NaL models; comparison between PAH group orientations in ligand/Gd complex for singlet ground state; optimized structures for the different rotamers of **P1** and **1**–**3** for the triplet states; the **Na1** optimized model; comparison between the spin density of protonated and deprotonated forms for all ligands and rotamers; comparison between spin density of protonated and deprotonated forms for all ligands (rotamer A); comparison between DFT ZFS parameters *D* and *E* for **P0**/**P1** and **1**–**3**, rotamer A; RO-BP86 Lüwdin (Mulliken) spin populations in % for the precursor **P1** and ligands **1**–**3** and in the NaL model (**NaP1** and **Na1**–**Na3**); RO-BP86 ZFS parameters *D* and *E* for all rotamers of optimized NaL models; and comparison between DFT ZFS parameters *D* and *E* for **P1** and **1**–**3** in protonated and deprotonated forms (rotamer A) (PDF)
