# Supplementary material for: Nature of the Ligand-Centered Triplet State in Gd3+ β-Diketonate Complexes as Revealed by Time-Resolved EPR Spectroscopy and DFT Calculations
Source: Inorg Chem. 2021 Oct 6;60(20):15141–50. doi: 10.1021/acs.inorgchem.1c01123 (PMC8763374; doi:10.1021/acs.inorgchem.1c01123)
Supplement: Supplementary file 1 — ic1c01123_si_001.pdf [file ic1c01123_si_001.pdf]

Supporting Information

The nature of the ligand-centered triplet state in Gd^{3+} β-diketonate complexes as revealed by Time-Resolved EPR spectroscopy and DFT calculations.

Silvia Carlotto,[1,2,*] Luca Babetto,[1] Marco Bortolus,[1,*] Alice Carlotto,[1] Marzio Rancan,[2] Gregorio Bottaro,[2] Lidia Armelao,[1,3] Donatella Carbonera[1] and Maurizio Casarin[1]

[1]Department of Chemistry, University of Padova, via F. Marzolo 1, 35131 Padova, Italy

[2]Institute of Condensed Matter Chemistry and Technologies for Energy (ICMATE), National Research Council (CNR), c/o Department of Chemistry, University of Padova, via F. Marzolo 1, 35131 Padova, Italy

[3]Department of Chemical Sciences and Technology of Materials (DSCTM), National Research Council (CNR), Piazzale A. Moro 7, 00185 Roma, Italy

Corresponding authors:

Silvia Carlotto, email: silvia.carlotto@unipd.it

Marco Bortolus, email: marco.bortolus@unipd.it

Table of contents:

TR-EPR Simulations (Table S1, Figure S1, S2)	Pages S3-S6
Figure S3	Page S7
Figure S4	Page S8
Figure S5	Page S8
Figure S6	Page S9
Figure S7	Page S10
Figure S8	Page S11
Figure S9	Page S12
Table S2	Page S13
Table S3	Page S14
Table S4	Page S15
Table S5	Page S16

TR-EPR simulations

Experimental and simulated Time-Resolved-EPR (TR-EPR) spectra of the ligands and Gd^{3+} complexes in frozen solution (80 K) are reported in Figure S1 and S2, respectively, while the parameters obtained from the simulations are collected in Table S1. The simulations were performed using the development version of EasySpin, version 6.0.0 – dev34. In greater detail, the ZFS parameters were estimated directly from the experimental spectra and simulations were performed to obtain the starting parameters (ZFS parameters, zero-field populations, relative abundance in the case of multiple species, gaussian anisotropic linewidths – as full width at half height) to be optimized. Then, the populations and relative abundance (and when needed the linewidths) were optimized using the Levenberg-Marquardt algorithm within EasySpin using the *esfit* function. Note that, before the final optimization, Monte Carlo fitting was run to explore the parameter space and to ensure that the starting parameters were not representative of a local minimum.

The TR-EPR spectra of the excited triplet states, unlikely conventional EPR spectra, are not displayed as first derivative lineshapes and can be either in emission or enhanced absorption (indicated by the E/A red arrows in Figures S1 and S2). The E/A intensities originate from the non-Boltzmann populations of the three triplet sublevels. The line shapes of all the TR-EPR spectra of **GdP1, Gd1** – **Gd3** are compatible with the formation of the triplet states via Intersystem Crossing (ISC) from the first excited singlet state. The TR-EPR spectrum of **GdP1** is dominated by one triplet species with EEE/AAA line shape. Although there are hints of a second triplet species with a larger $|D|$ ZFS parameter, this second species is too weak to be fully identified and simulated (<5% of relative abundance) and only its width (i.e. the Z component) is visible (green bands in Figure S2). The spectra of **Gd1** and **Gd2** are characterized by two triplet species, each one characterized by different ZFS parameters and polarization patterns (Figure S2). The spectrum of **Gd3** shows only a single triplet state with a AAA/EEE line shape. The widest visible species, highlighted by the green bands in Figure S2, is present in **GdP1** and **Gd1**, while the spectra narrow in **Gd2** and significantly in **Gd3**.

Table S1. Triplet parameters obtained from the simulations of the TR-EPR spectra, when two species are present each row reports two sets of parameters. Absolute values of the ZFS parameters |*D*| and |*E*| (cm^{-1}); |*E/D*| ratio; triplet sublevels population (P_x, P_y and P_z); relative amount of each spectral component (%); anisotropic linewidths (Lw_x, Lw_y, Lw_z in Mhz). The g tensor, collinear with the D tensor, is g_{xx}=2.006, g_{yy}=g_{zz}=2.009 for all compounds. n.d. = not determined.

	|*D*|	|*E*|	|*E/D*|	$P_x : P_y : P_z$	%	$Lw_x : Lw_y : Lw_z$ /Mhz
**P0**	0.111	0.030	0.270	0.80 : 0.00 : 0.20	100	50 : 180 : 50
**P1**	0.098	0.019	0.194	0.00 : 0.49 : 0.51	>95	10 : 230 : 230
	0.111	n.d.	n.d.	n.d.	n.d.	n.d.
**GdP1**	0.098	0.019	0.194	0.00 : 0.39 : 0.61	>95	45 : 250 : 50
	0.111	n.d.	n.d.	n.d.	n.d.	n.d.
**1 & Gd1**	0.092	0.017	0.185	0.00 : 0.00 : 1.00	63	150 : 154 : 180
	0.111	0.013	0.117	1.00 : 0.00 : 0.00	37	155 : 186 : 180
**2**	0.090	0.008	0.089	0.07 : 0.00 : 0.93	62	100 : 100 : 100
	0.100	0.024	0.240	0.93 : 0.07: 0.00	38	150 : 50 : 50
**Gd2**	0.090	0.008	0.089	0.00 : 0.02 : 0.98	73	200 : 50 : 200
	0.100	0.024	0.240	0.90 : 0.00 : 0.10	27	50 : 100 : 100
**3**	0.074	0.016	0.216	0.79 : 0.21 : 0.00	100	10 : 21 : 10
**Gd3**	0.070	0.016	0.229	0.49 : 0.51 : 0.00	100	50 : 50 : 50

Figure S1. Left. Time-resolved EPR experimental (black lines) spectra and simulations (red dotted lines) (λ_{exc} = 355 nm) of the precursors **P0** and **P1** and ligands **1 – 3** in frozen toluene solution at X-band (microwave frequency ν = 9.705 GHz), T = 80 K. Right. The normalized individual components of the simulations (broader component blue, narrower green). The green side-bands correspond to the maximum width of the spectra of **P0**, **P1**, **1** (equal to 2|*D*|*h/gμ_B) and highlight the progressive narrowing of the EPR spectra along the series. The red arrows denote the signals in enhanced absorption (A) and emission (E).

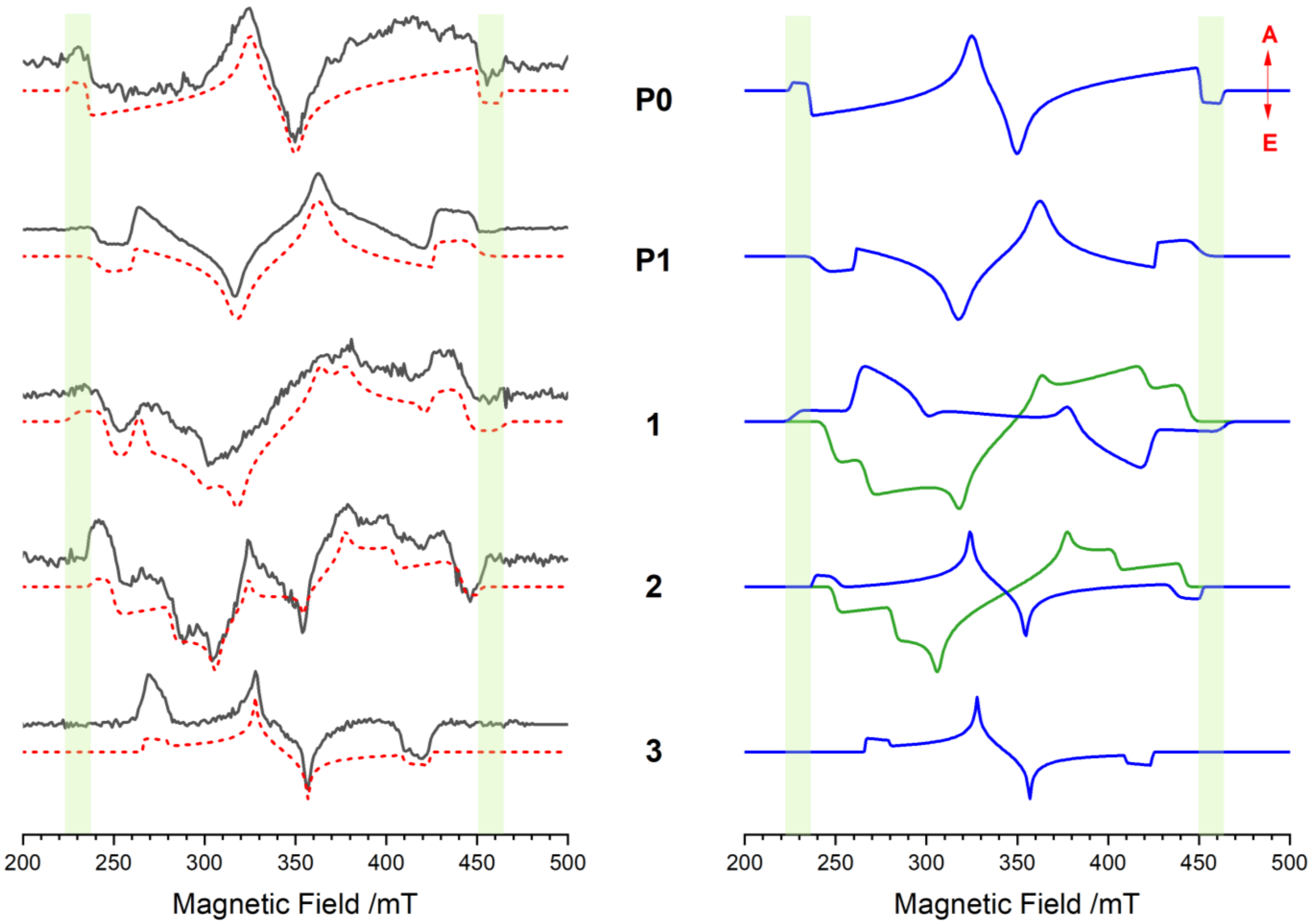

Figure S2. Left. Time-resolved EPR experimental (green lines) spectra and simulations (red dotted lines) (λ_{exc} = 355 nm) of complexes **GdP1**, **Gd1 – Gd3** in frozen toluene solution at X-band (microwave frequency ν = 9.705 GHz), T = 80 K. Right. The normalized individual components of the simulations (broader component blue, narrower green). The green side-bands correspond to the maximum width of the spectra of **GdP1** and **Gd1** (equal to 2|*D*|*h/$g_{\mu B}$) and highlight the progressive narrowing of the EPR spectra along the series. The red arrows denote the signals in enhanced absorption (A) and emission (E).

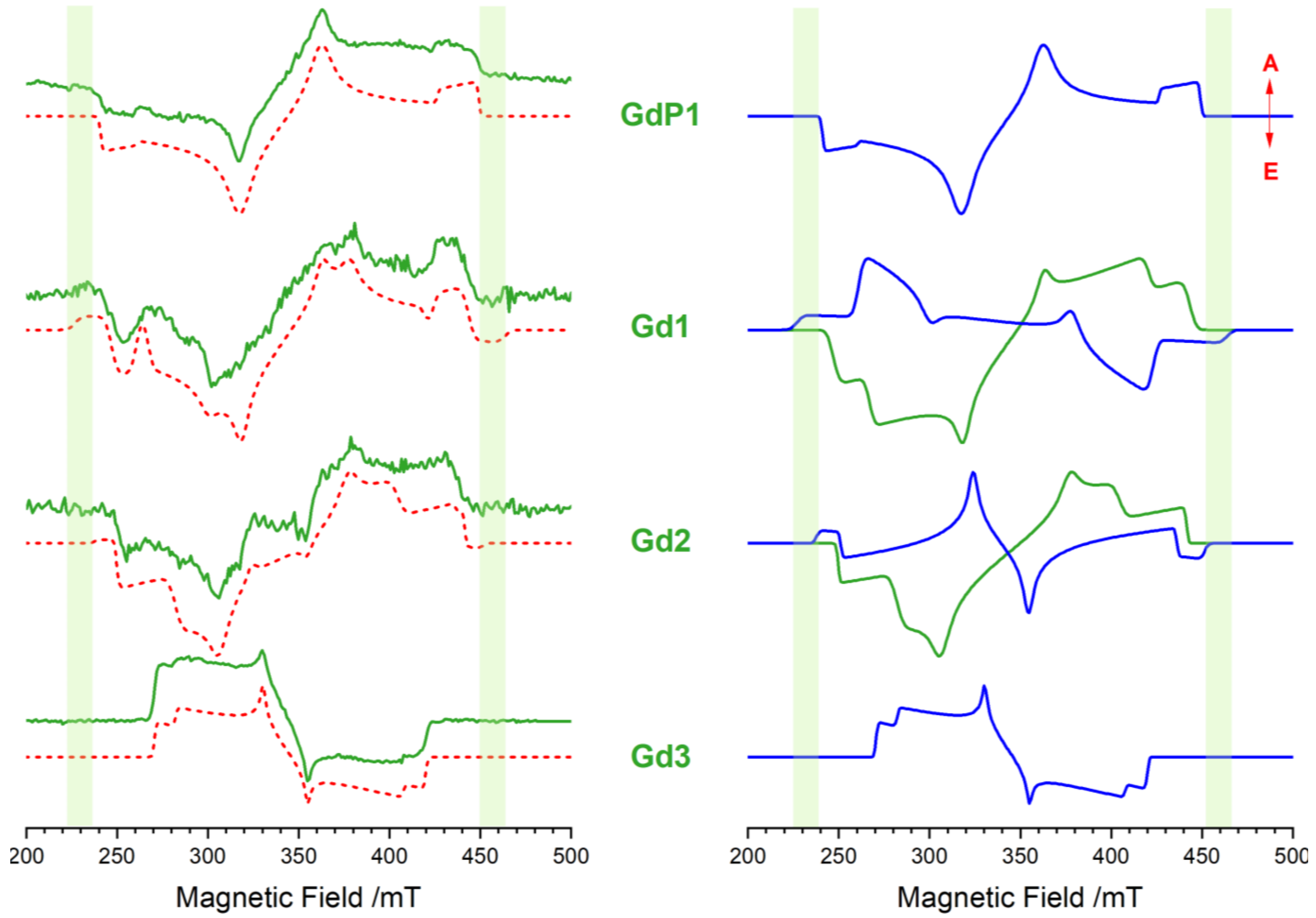

Figure S3. Adsorption spectra for the precursor **P1** and ligands **1** - **3**. The vertical dotted line at 355 nm is the λ_{exc} used in the Time-resolved EPR spectra.

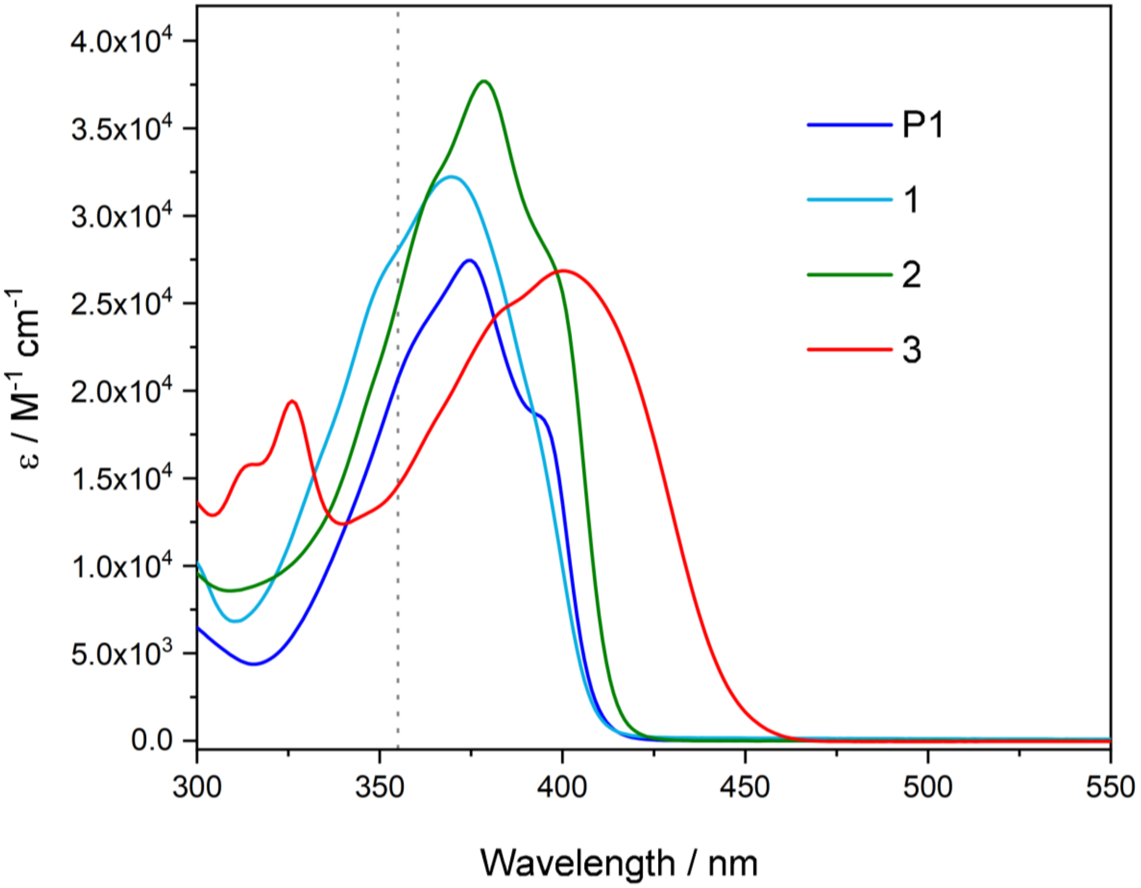

Figure S4. ZFS principal axes system in the molecular frame as obtained by DFT calculations. Top row shows the precursors and the neutral ligands, bottom row the deprotonated systems with a sodium atom as a counterion. Grey, white, yellow, violet, and red spheres are C, H, S, Na and O atoms, respectively.

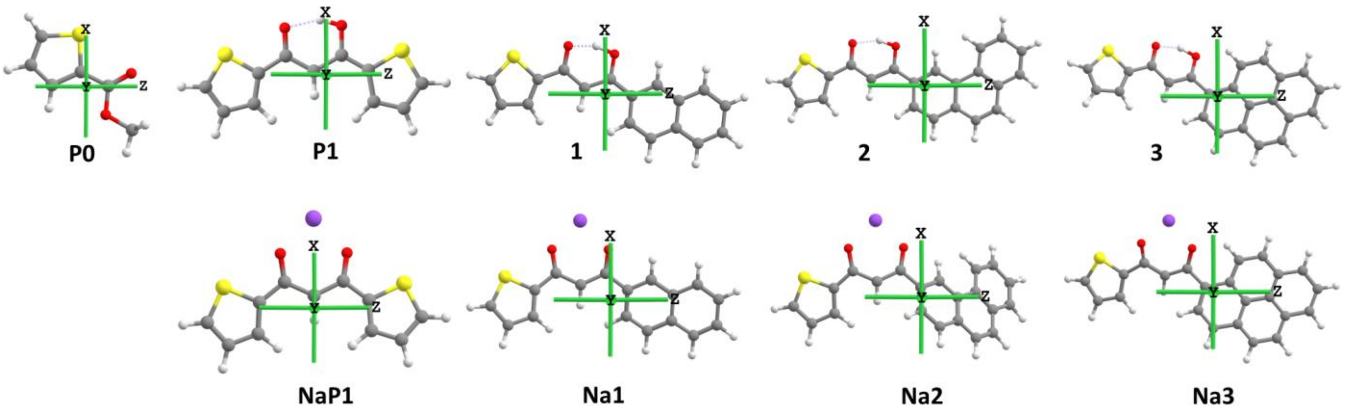

Figure S5. Comparison between thienyl or PAH group orientations in precursor/Gd complexes and ligands/Gd complexes (singlet ground states). Grey, yellow, red and green spheres are C, S, O and La atoms, respectively. H atoms are omitted for clarity. Dihedral angles are given in deg.

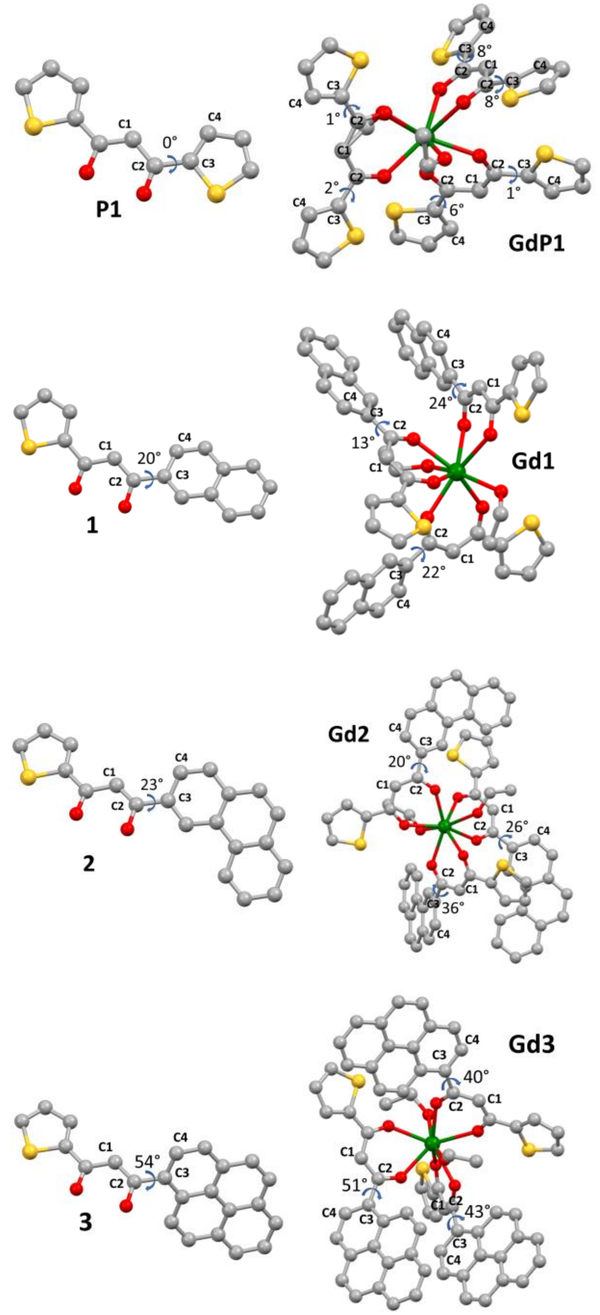

Figure S6. Optimized structures for the different rotamers of **P0**, **P1** and **1 - 3** calculated at the PBE0 level for the triplet states. Grey, white, yellow, and red spheres are C, H, S and O atoms, respectively. The energy differences Δ (kcal/mol) are given with respect to the most stable form.

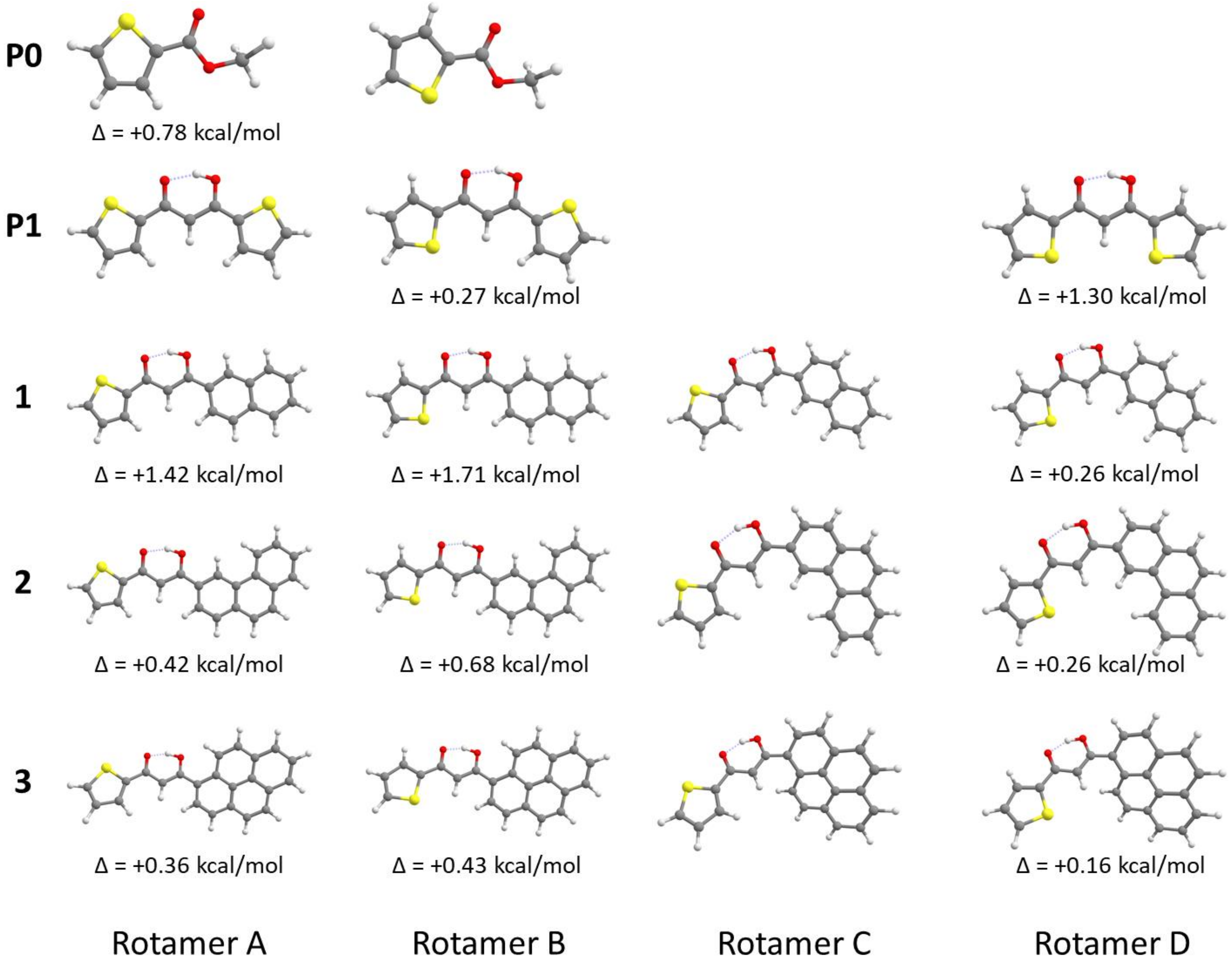

Figure S7. The **Na1** optimized model. Grey, white, yellow, violet, and red spheres are C, H, S, Na and O atoms, respectively. The O-Na distance is given in Å.

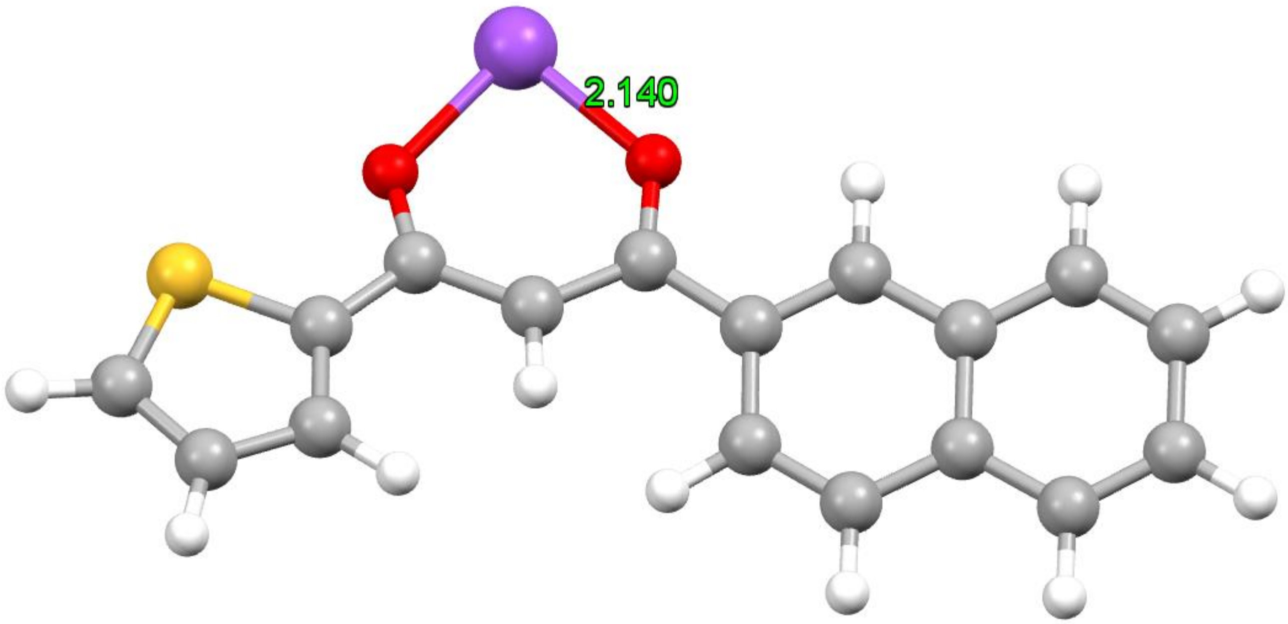

Figure S8. Comparison between spin density of protonated and deprotonated forms for the precursor **P1** and all ligands, considering all and rotamers, calculated at the RO-BP86 level. Displayed isosurfaces correspond to 0.003 $e^{1/2}$ x $Å^{-3/2}$ values. Grey, white, yellow, violet, and red spheres are C, H, S, Na and O atoms, respectively.

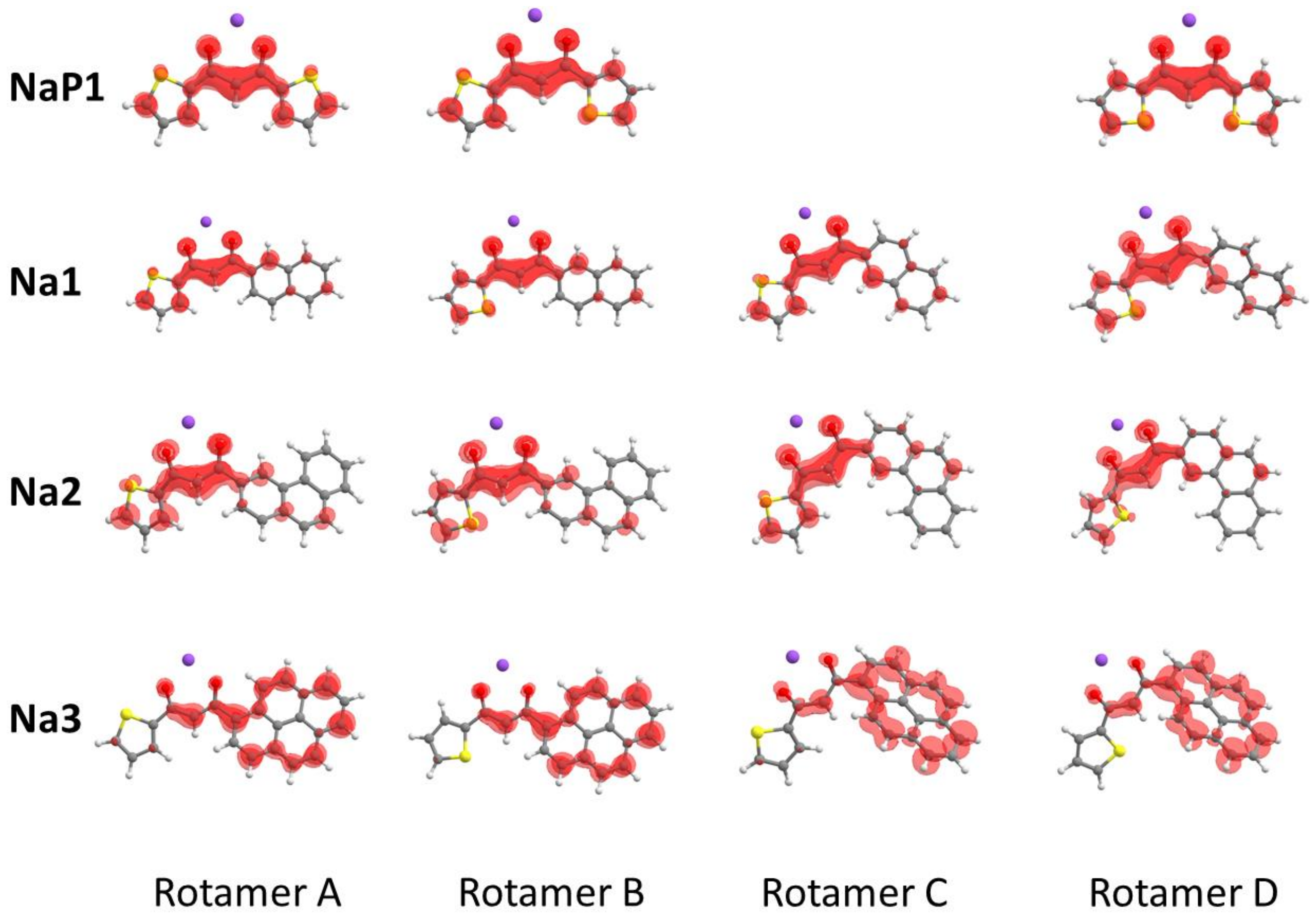

Figure S9. Comparison between spin density of protonated and deprotonated forms for the precursor **P1** and all ligands (rotamer A), calculated at the RO-BP86 level. Displayed isosurfaces correspond to 0.003 $e^{1/2} \times Å^{-3/2}$ values. Grey, white, yellow, and red spheres are C, H, S and O atoms, respectively.

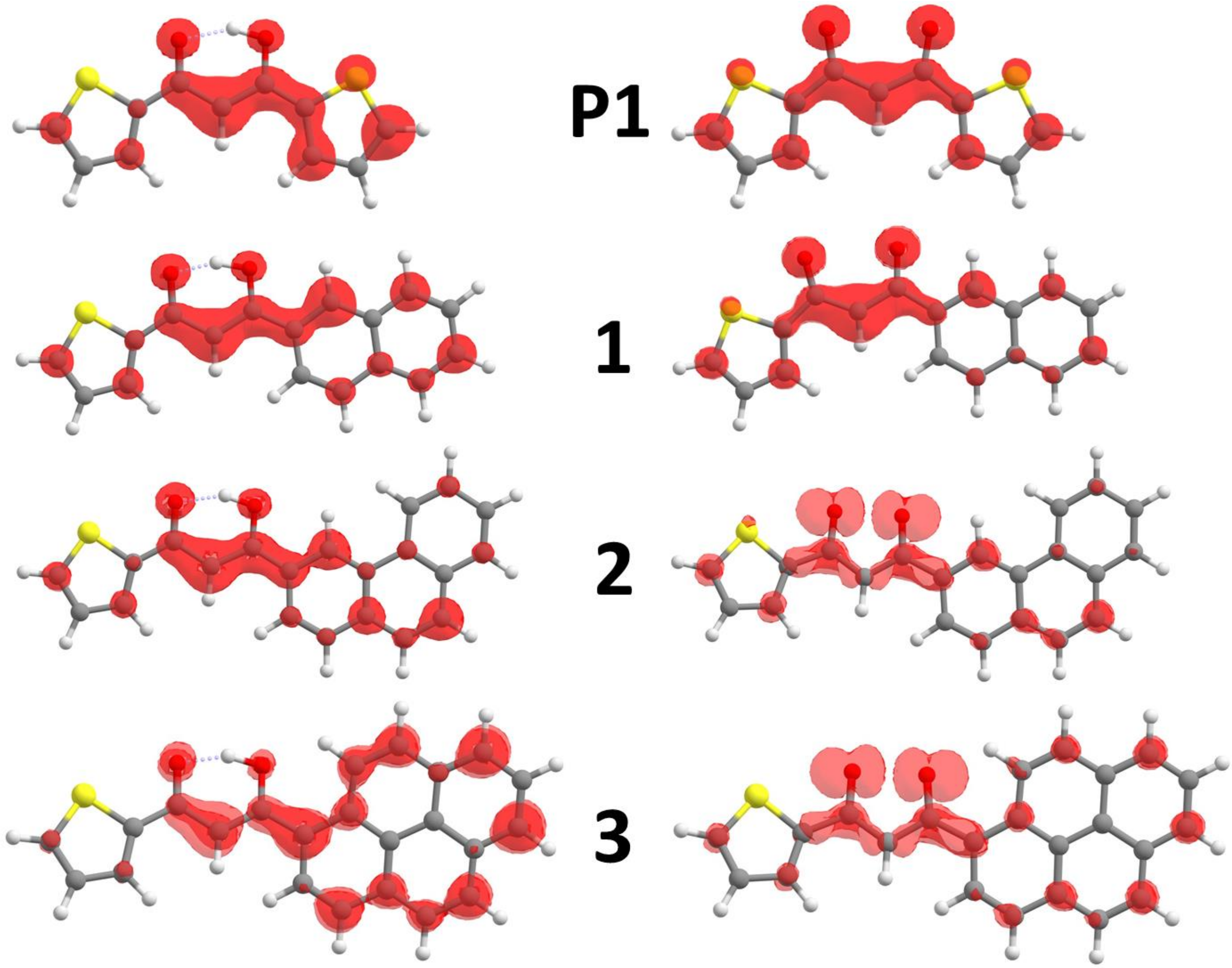

Table S2. Comparison between DFT ZFS parameters *D* and *E/D* for **P0**, **P1** and **1** - **3**, rotamer A. *D* and *E* parameters are given in cm^{-1}.

	RO-BP86		RO-B3LYP		Experimental	
	D	*E/D*	*D*	*E/D*	|*D*|	|*E/D*|
**P0**	-0.070	0.329	-0.068	0.324	0.111	0.270
**P1**	-0.067	0.254	-0.068	0.191	0.098	0.194
**1**	-0.071	0.085	-0.080	0.063	0.092	0.185
**2**	-0.070	0.100	-0.080	0.075	0.090	0.089
**3**	-0.039	0.103	-0.038	0.132	0.074	0.216

Table S3. RO-BP86 Löwdin spin populations (Mulliken in parenthesis) in % for the precursor **P1**, ligands **1** - **3** and in the NaL models (**NaP1, Na1 – Na3**). % have been calculated by summing the individual atomic spin population contribution for each fragment (Thiophene, Diketone, PAH fragments). The different fragments are shown in figure below.

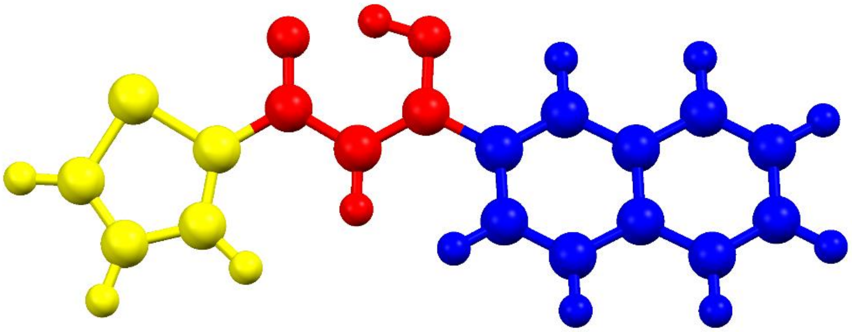

	Thiophene (yellow)	Diketone (red)	PAH (blue)
**P1**	10.2% (9.9%)	50.5% (51.1%)	39.3% (39.0%)[a]
**1**	12.3% (12.0%)	43.9% (44.4%)	43.8% (43.6%)
**2**	9.6% (9.3%)	45.9% (46.6%)	44.5% (44.1%)
**3**	4.1% (4.0%)	21.7% (21.3%)	74.2% (71.7%)
**NaP1**[b]	20.4% (20.0%)	59.2% (60.1%)	20.4% (19.9%)
**Na1**[b]	21.0% (20.6%)	60.6% (61.5%)	18.4% (17.9%)
**Na2**[b]	22.0% (21.5%)	58.2% (59.2%)	19.8% (19.3%)
**Na3**[b]	3.6% (3.5%)	19.3% (19.0%)	77.1% (77.5%)

[a] The PAH group in **P1** is the thiophene group in the enol side.

[b] In the NaL models, the Na atom is included in the diketone fragment

Table S4. RO-BP86 ZFS parameters *D* and *E/D* for all rotamers of optimized NaL models (**NaP1, Na1 – Na3**). *D* and *E* parameters are given cm^{-1}. Absolute experimental values are reported for Gd^{3+} complexes (**GdP1**, **Gd1 – Gd3**).

	Rotamer A		Rotamer B		Rotamer C		Rotamer D		Experimental	
	D	*E/D*	*D*	*E/D*	*D*	*E/D*	*D*	*E/D*	|*D*|	|*E/D*|
**NaP1**	-0.078	0.137	-0.081	0.125	//	//	-0.086	0.119	0.098	0.194
**Na1**	-0.080	0.114	-0.085	0.092	-0.070	0.153	-0.075	0.112	0.092	0.185
**Na2**	-0.072	0.097	-0.076	0.071	-0.069	0.142	-0.073	0.095	0.090	0.089
**Na3**	-0.040	0.118	-0.039	0.118	-0.037	0.149	-0.036	0.156	0.070	0.228

Table S5. Comparison between DFT ZFS parameters *D* and *E/D* and the absolute experimental values for **P1, 1 - 3** in protonate and deprotonate forms (rotamer A). *D* and *E* parameters are given in cm^{-1}.

	Protonated		Deprotonated		Experimental	
	D	*E/D*	*D*	*E/D*	|*D*|	|*E/D*|
**P1**	-0.067	0.209	-0.068	0.176	0.098	0.194
**1**	-0.071	0.085	-0.063	0.143	0.092	0.185
**2**	-0.070	0.100	-0.057	0.070	0.090	0.089
**3**	-0.039	0.128	0.169	0.071	0.074	0.216
